# From symptom scales to regulatory endpoints: the evolution and clinical impact of patient-reported outcome measures in myeloproliferative neoplasms

**DOI:** 10.1007/s10238-025-01830-9

**Published:** 2025-10-25

**Authors:** Meng Chen, Chengyulong Zheng, Juan Xie, Weifeng Zhang, Ying Zhang

**Affiliations:** 1 Department of Hematology, First Teaching Hospital of Tianjin University of Traditional Chinese Medicine, 88 Changling Road, Xiqing District, Tianjin, 300381 China; 2 National Clinical Research Center for Chinese Medicine Acupuncture and Moxibustion, 88 Changling Road, Xiqing District, Tianjin, 300381 China

**Keywords:** Myeloproliferative neoplasms, Symptom burden, Patient-reported outcome measures, Symptom assessment form, Regulatory approval, Clinical trial

## Abstract

Myeloproliferative neoplasms (MPNs) are symptom-driven hematologic malignancies characterized by persistent and heterogeneous symptom burden that significantly impairs health-related quality of life (HRQoL). This burden is intrinsically linked to MPN pathophysiology, including splenomegaly, inflammatory cytokine release, and microvascular dysfunction, underscoring the need for MPN-specific patient-reported outcome measures (PROMs) to quantitatively assess symptoms and sensitively capture treatment responses. Initial instruments such as the Myelofibrosis Symptom Assessment Form (MF-SAF) and MPN Symptom Assessment Form (MPN-SAF) evaluated both symptom burden and HRQoL. To meet regulatory standards for JAK inhibitor trials, subsequent versions, such as MF-SAF v2.0 and the MPN-SAF Total Symptom Score (TSS), shifted focus toward symptom-specific endpoints, with a ≥ 50% reduction in TSS (TSS50) considered a clinically meaningful response. To improve consistency and methodological rigor, the MF-SAF was further refined into version 4.0, which has served as a primary endpoint in pivotal trials such as MOMENTUM, often in combination with validated generic HRQoL instruments. These PROMs have played a pivotal role in securing regulatory approvals for agents such as ruxolitinib and momelotinib. While TSS50 remains the standard endpoint in clinical trials, its dichotomous nature presents limitations; emerging evidence suggests that evaluating TSS as a continuous variable may offer greater sensitivity in capturing treatment effects. In clinical practice, the MPN-SAF TSS is increasingly used to guide symptom monitoring and personalized decision-making. This review outlines the evolution, validation, and clinical impact of MPNs-symptom-specific PROMs, underscoring their growing role in delivering precision, patient-centered care.

## Introduction

In the evolving landscape of precision medicine and patient-centered care, the evaluation of health outcomes has expanded beyond conventional laboratory and imaging findings. Patient-reported outcomes (PROs), defined by the U.S. Food and Drug Administration (FDA) as direct reports from patients about their health condition without interpretation by clinicians or others, have emerged as indispensable tools for quantifying these subjective yet impactful aspects of disease. The instruments used to collect PRO data, known as patient-reported outcome measures (PROMs), allow for structured, validated, and reproducible assessment of symptoms, functional status, and health-related quality of life (HRQoL). PROMs are essential for fully capturing symptom burden, evaluating early therapeutic responses, and aligning with patient priorities [1].

Philadelphia chromosome-negative myeloproliferative neoplasms (Ph-negative MPNs, hereafter MPNs)—including polycythemia vera (PV), essential thrombocythemia (ET), primary myelofibrosis (PMF), and post-PV/ET myelofibrosis (MF) [2]—are chronic hematologic malignancies driven predominantly by dysregulated Janus kinase (JAK)-signal transducer and activator of transcription signaling [3]. The aberrant activation underlying MPNs gives rise to a biologically distinct and burdensome symptom profile: splenomegaly-related symptoms (e.g., abdominal discomfort, early satiety and mechanical pressure), constitutional symptoms mediated by inflammatory cytokines (e.g., fatigue, pruritus and night sweats) and microvascular dysfunction (e.g., headache, dizziness, and cognitive impairment) [4]. These symptoms are persistent, heterogeneous, and profoundly impact HRQoL [5, 6], establishing MPNs as prototypical examples of symptom-driven malignancies.

Generic symptom assessment tools such as Brief Fatigue Inventory (BFI) [7] or Functional Assessment of Cancer Therapy (FACT)-anemia (FACT-An) [8] lacked the ability to reflect hallmark MPNs symptoms, particularly those related to cytokine dysregulation and splenic enlargement. HRQoL instruments such as the European Organization for Research and Treatment of Cancer (EORTC) quality-of-life questionnaire-core 30 (QLQ-C30) [9], although broad in scope, often dilute the detection of symptom-specific changes and are insufficiently sensitive to clinical manifestations. The biologically driven nature of these symptoms highlights the need for disease-specific PROMs that are both biologically relevant and psychometrically robust. This review synthesizes the scientific evolution of MPNs-symptom-specific PROMs from early symptom scales to their current role as regulatory-grade endpoints. It explores their development, clinical and regulatory application, and real-world integration. Special attention is given to the necessity of aligning PROMs design with MPNs pathophysiology, as well as to future opportunities and challenges in advancing patient-centered assessment strategies in this field.

## The clinical relevance of PROMs in MPNs

### Symptom burden and heterogeneity in MPNs

The symptomatic landscape of MPNs is defined by its complexity and variability, underscored by early landmark studies. A 2007 internet-based survey of 1179 patients revealed that 80.7% reported fatigue, 52.2% pruritus, and 49.2% night sweats, with symptoms causing substantial medical disability [4].

Fatigue, a hallmark symptom among cancer patients, arises from multiple factors, including tumor burden, treatment toxicity, pain, and anemia [10]. Although not specific to MPNs, fatigue is notably more prevalent and severe in this population, as measured by tools such as the BFI [7] and FACT-An [8].

Large-scale observational studies have further characterized this symptom burden. For example, the MPN Landmark survey revealed that 90% of patients reported disease-related symptoms within 12 months of diagnosis, even those with low-risk disease, challenging traditional risk stratification that prioritizes hematologic parameters over patient experience [11]. Moreover, the survey showed that physicians significantly underestimated the proportion of symptomatic PV or ET patients at diagnosis compared to patient reports [12]. Furthermore, while patients prioritized symptom relief as a major treatment goal, physicians tended to prioritize disease control [12].

The German Study Group MPN Bioregistry (GSG-MPN), which included 3979 patients, confirmed that self-reported symptoms using MPN symptom assessment form (MPN-SAF) total symptom score (TSS) [13] were significantly higher than those recorded by physicians, with no improvement in symptom recognition over time [14]. This discordance underscores the critical role of PROMs in aligning clinical assessments with the patient experience.

Symptom burden also exhibits substantial heterogeneity across different MPNs subtypes. PMF patients generally report the highest symptom severity [4, 5, 15], driven by factors such as splenomegaly, systemic inflammation, and ineffective hematopoiesis. A Quebec MPN Research Group [16] used the MPN-SAF TSS to assess symptom burden and found that MF patients exhibited significantly higher mean and peak symptom scores for fatigue, early satiety, and bone pain compared to those with PV or ET. Notably, 14% of MF patients had MPN-SAF TSS scores greater than 40, compared to 6% in PV and 4% in ET. In contrast, GSG-MPN data suggested more comparable symptom burden across MPNs subtypes, possibly reflecting cohort differences or lower symptom severity in their MF group [14].

PV and ET patients demonstrated comparable overall symptom scores. PV patients reported more pruritus [16]. Gender and age disparities also emerged, with younger females reporting higher symptom scores [16], a trend supported by previous studies [4, 17, 18]. In MF, symptom reporting was less influenced by gender, likely due to male predominance and comorbidities [17], which further complicates the symptom landscape.

Temporal variability also adds complexity. In a 1443-patient cohort, symptoms such as concentration difficulties, insomnia, night sweats, and declines in QoL worsened over time, while others remained stable [19].

Collectively, the high symptom burden and marked heterogeneity highlight the indispensable role of standardized PROMs like MPN-SAF TSS. These tools are essential for accurately quantifying patient experiences, monitoring disease impact, and guiding patient-centered therapeutic strategies.

### Impact of symptom burden on HRQoL

While MPNs patients, especially PV and ET generally have favorable long-term survival [20], persistent symptom burden continues to significantly affect HRQoL.

The MPN Landmark survey was the first large observational study to evaluate the impact of MPNs on daily living and work productivity. It found considerable functional impairment even in patients without disease progression [11]. These findings have been corroborated by additional studies [6, 21]. Similarly, the REVEAL study demonstrated that PV patients face substantial QoL deterioration, as measured by tools like the MPN-SAF and QLQ-C30 [22]. The myelofibrosis and essential thrombocythemia observational study found that both low-risk MF and low- or high-risk ET patients experience a significant symptom burden, as assessed by the MPN-SAF TSS, which negatively affect.

QoL, measured by the QLQ-C30 and work ability evaluated by the work productivity and activity impairment questionnaire-specific health problem [23].

Thus, this paradox of “prolonged survival but profound suffering” highlights the urgent need for PROMs to assess non-hematologic outcomes, enabling clinicians to fully appreciate the subjective disease burden and true benefits of treatment.

### Symptom burden and HRQoL as prognostic indicators

Severe symptom burden, particularly constitutional symptoms such as fever, night sweats, weight loss [24] has been consistently recognized as a negative prognostic indicator in MF. Major prognostic models, including the International Prognostic Scoring System (IPSS) [25], the dynamic IPSS (DIPSS) [26], its refined version DIPSS-Plus [27], and Mutation-Enhanced IPSS [28], all incorporate the presence of constitutional symptoms as a key adverse factor.

A study by Bankar et al. involving 439 chronic MF patients found that higher frailty scores, as evaluated by a 35-item frailty index, were associated with reduced survival and increased risk of JAK inhibitor treatment failure [29]. The GSG-MPN analysis [15] identified severe fatigue and weight loss as factors significantly associated with increased mortality risk in MPNs patients, after adjusting for age, sex, and disease duration.

While some smaller cohorts, such as a study of Thai MPNs patients did not observe significant correlations between MPN-SAF TSS score and survival [30]. Larger studies such as the Quebec MPN cohort linked high symptom burden (MPN-SAF TSS ≥ 35) to worse overall survival (OS) in PV. However, similar effects were not observed in ET or MF [16]. Notably, in PV, high symptom burden was also associated with an increased risk of fibrotic transformation. In MF, baseline QoL independently predicted OS in the COMFORT-1 trial [31].

### Development and validation of PROMs in MPNs

While often used interchangeably, PROs and HRQoL represent related yet distinct constructs. PROs encompass all direct patient-reported health information, including symptoms, functional status, treatment tolerability, and adverse events, without clinician interpretation. In contrast, HRQoL is a subdomain of PROs focusing on “the functional effect of an illness and its consequent therapy upon a patient, as perceived by the patient” [32].

In MPNs, the selection of appropriate PROMs depends on the measurement objective, such as assessing overall health status, individual symptom burden, or supporting regulatory and economic evaluations. PROMs used in MPNs may be broadly categorized into three domains: multidimensional cancer-specific HRQoL tools, utility-oriented generic instruments, and single-symptom or MPNs- symptom-specific tools.

### Multidimensional cancer-specific HRQoL instruments

The QLQ-C30 [8] is the most widely used cancer-specific HRQoL instrument in MPNs research. It comprises 30 items across five functional domains (physical, role, emotional, cognitive, and social), nine symptom scales (e.g., fatigue, nausea/vomiting, pain), and a global QoL score. It provides comprehensive assessment of disease impact. QLQ-C30 has been used in key trials such as COMFORT-I [33] and MOMENTUM [34], and its scores can be mapped to EQ-5D utility values for health economic modeling [35]. The Myelofibrosis 8 dimensions, developed from QLQ-C30 and MF-SAF data, thus facilitates MF-specific economic evaluations [36].

The Hematological Malignancy-Specific Patient-Reported Outcome (HM-PRO) is the first generic hematological malignancy-specific PRO measure for use in clinical practice and clinical trial [37]. It integrates symptom and HRQoL domains and has been validated in over 900 patients, including those with MPNs, demonstrating strong psychometric performance. Although its application in MPNs-specific trials remains limited, its cross-platform compatibility—available in both paper and electronic formats—supports broad implementation as an ePRO tool [38]. Cross-cultural validation studies, such as the Danish adaptation, affirm its suitability for routine clinical use and patient engagement in hematologic care [39].

The Patient-Reported Outcomes Measurement Information System (PROMIS), developed by the National Institutes of Health, includes 124 item banks spanning physical, mental, and social health domains [40]. PROMIS fatigue [41]—a 7-item instrument with a 7-day recall—is gaining attention in MPNs research for its reliability and adaptability [35, 55]. PROMIS Short Form 10b has also been employed in the MOMENTUM trials [34], offering a concise yet comprehensive HRQoL profile.

### Utility-oriented generic HRQoL tools

EQ-5D, developed by EuroQol, is a preference-based, generic HRQoL instrument comprising five domains and available in 3-level (3L) and 5-level (5L) versions [42]. Paired with the EQ Visual Analog Scale (EQ-VAS)—a 0–100 self-rating of overall health—EQ-5D enables calculation of health utility indices and quality-adjusted life years. It has been used in JAKARTA (EQ-5D-3L) [43] and MOMENTUM (EQ-5D-5L) [34], and shown to detect health gains in low-risk patients in the ROMEI study [44].

The Short Form 36-Item Health Survey (SF-36) and its abbreviated version, SF-12 [45] assessing eight health domains. Post hoc analysis from SIMPLIFY trials of the momelotinib demonstrated that transfusion-dependent patients had lower SF-36 scores, with transfusion independence correlating with most domain improvements [46].

The Patient Global Impression of Change (PGIC) is a single-item global scale that uses a seven-point scale to assess a patient’s subjective perception of improvement since initiating treatment. In COMFORT-I trial [33], PGIC closely aligned closely with TSS and fatigue improvements. In MANIFEST, PGIC served as an anchor PRO for TSS changes, with each one-category PGIC deterioration associated with an 11–12% higher TSS, validating its value as an anchor to interpreting clinical meaningfulness [47].

### Symptom-specific and MPNs-symptom-specific tools

Single-symptom PROMs, available in both single-item and multi-item formats, are commonly used in MPNs to evaluate core symptoms, particularly fatigue. Single-item scales (e.g., a 0–10 fatigue severity rating) offer simplicity and practicality for rapid screening in clinical settings, but lack of dimensionality specificity. This limits their utility in distinguishing components like physical versus cognitive fatigue or capturing subtle changes over time. In contrast, multi-item instruments offer structured insight into both intensity and interference with daily function. The BFI [7], for example, assesses fatigue intensity and its impact on daily activities. The FACT system offers a modular framework for evaluating HRQoL. Its core instrument, FACT-G, comprises 27 items spanning physical, social/family, emotional, and functional well-being domains [48]. The FACT-F module supplements FACT-G with 13 fatigue-specific items, enabling comprehensive evaluation of fatigue across multiple dimensions [8] and remains widely utilized in oncology research. FACT-An [7], which combines FACT-F with seven additional items addressing anemia-related issues, is specifically tailored for populations with anemia-related symptom burden. In a cohort of 85 MF patients, those who responded to anemia-directed therapy showed marked and sustained FACT-An improvement from week 4 onward [49], highlighting its relevance for fatigue- and anemia-predominant populations.

**Fig. 1:**
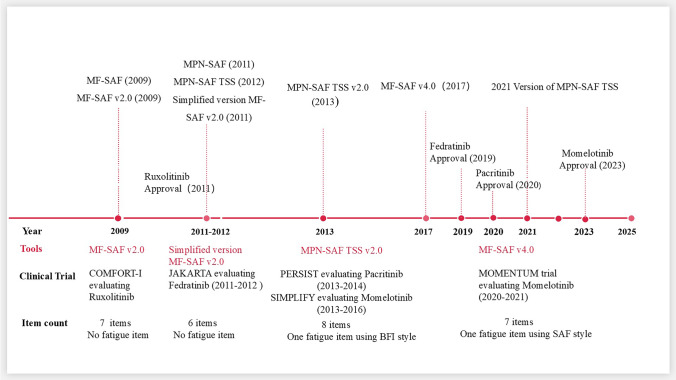
Timeline of symptom-specific patient-reported outcome measures evolution and JAK kinase inhibitors approvals. Abbreviations: MF-SAF, Myelofibrosis Symptom Assessment Form; MPN-SAF, Myeloproliferative Neoplasms Symptom Assessment Form; TSS, Total Symptom Score; BFI, Brief Fatigue Inventory;

In contrast to generic tools, MPNs-symptom-specific PROMs are designed to capture the disease’s unique pathophysiologic manifestations. The development typically involves: (1) conceptual framework development through literature reviews, patient interviews, and expert input; (2) item generation and cognitive testing to ensure content validity; and (3) pilot testing to refine structure and optimize psychometric performance. Earlier tools like the MF-SAF [50] and MPN-SAF [51] initially included a global QoL item. To meet regulatory guidelines from the U.S FDA, later versions (e.g., MF-SAF v2.0 [33] and its modified versions) utilized in JAK inhibitor clinical trials excluded the QoL items and enhanced internal consistency to emphasize symptom-specific assessment. The Consensus-based Standards for the selection of health measurement instruments (COSMIN) framework recommends that validated PROMs demonstrate robust psychometric properties, including: content validity, structural validity (e.g., confirmatory factor analysis with comparative fit index ≥ 0.95, root mean square error of approximation ≤ 0.06), internal consistency (Cronbach’s α ≥ 0.70) and test–retest reliability (intraclass correlation coefficient [ICC] ≥ 0.70 acceptable, ≥ 0.90 excellent. Together, these standards ensure scientific robustness and clinical meaningfulness for both research and routine care. Additionally, cross-cultural validity and low measurement error, quantified through the standard error of measurement and smallest detectable change, are essential for ensuring consistency across settings. Critically, the validated MF-SAF v4.0 [52] was developed and used as primary endpoints in pivotal trials such as MOMENTUM [34], supporting FDA approvals of momelotinib and demonstrating their regulatory impact. As shown in Fig. 1, a timeline illustrating the evolution of symptom-specific PROMs in clinical trials of JAK inhibitors is presented alongside the approval dates of JAK kinase inhibitors. MPNs trials now often combine disease-specific PROMs (e.g., MF-SAF v4.0 [52]) and multidimensional HRQoL tools like QLQ-C30, allowing detailed symptom monitoring alongside comprehensive HRQoL assessment. This integration not only reflects the scientific progression of MPNs-symptom-specific PROMs development but also enhances the clinical utility of trials by addressing both specific symptoms and overall patient well-being.

### MPNs-symptom-specific PROMs

#### Early development of MF-SAF: a symptom-centered breakthrough

The original MF-SAF [50], developed in 2009, was the first disease-specific instrument designed to systematically assess the symptom burden in patients with MF. Derived from an international internet-based survey of 458 patients with MF, this 20-item instrument included 18 symptom items, a single-item QoL rating, and an open-ended question. Among the 18 symptom items, nine assessed fatigue severity and interference using the BFI framework, while the remaining nine targeted symptoms specific to MF, which were reported by more than 10% of respondents. These included four splenomegaly-associated symptoms (e.g., early satiety, abdominal discomfort, cough, and inactivity) and five systemic inflammation-related symptoms (e.g., night sweats, pruritus, bone pain, fever, and unintentional weight loss). Fatigue, recognized as one of the most burdensome symptoms in MF, was assessed using the BFI, a validated 9-item scales originally developed for oncology populations (3 severity items, 6 interference items; 0–10 numeric rating scale [NRS]) [7]. A total BFI score is calculated as the mean of the nine items. The BFI assesses the peak fatigue severity over the previous 24 h, while other items measure average symptom severity without a specification of recall period. Fever and weight loss were recorded as binary (Yes/No) items, while most other items used a 0–10 NRS, anchored at “Absent” (0) and “Worst Imaginable” (10). The global QoL item was similarly rated from 0 (“as good as it can be”) to 10 (“as bad as it can be”). An open-ended item enhanced comprehensiveness by capturing unstructured patient concerns.

As a hybrid instrument combing structured symptom scoring, global QoL evaluation, and open-ended patient input, the 2009 MF-SAF [50] provided a comprehensive overview of MF-related symptomatology. It was validated in early ruxolitinib trials for feasibility, responsiveness, and clinical utility in capturing patient-reported symptom experiences [53].

#### Regulatory-oriented refinement: MF-SAF v2.0

To align with FDA regulatory standards, MF-SAF [50] was revised in 2011 as MF-SAF v2.0 specifically for the phase III COMFORT-I trial (ruxolitinib vs placebo) [33]. Key modifications improved internal consistency and regulatory acceptability. Notably, the BFI-derived fatigue item was excluded: whereas other MF-SAF items measured the average symptom severity, the 9-item BFI assessed peak severity of fatigue. This discrepancy threatened the instrument’s coherence.

Additional exclusions included general systemic symptoms such as fever and unintentional weight loss, due to their limited variability over short intervals and suboptimal detectability via patient self-report. The global QoL item was also excluded to sharpen the focus on symptom-specific assessment. All remaining items were standardized to a 24-h recall period, enhancing consistency and compatibility with electronic PRO systems. The revised MF-SAF v2.0 retained seven core symptoms—abdominal discomfort, pain under the ribs on the left side, feeling full quickly (early satiety), night sweats, itching (pruritus), muscle/bone pain, and inactivity. Each was assessed using a 0–10 NRS. In COMFORT-I, TSS was calculated by summing six of the item scores (excluding inactivity), using the mean of daily scores over 28 days prior to week 24.

However, the cut-off value defining “significant” improvement warrants further validation, as its clinical sensitivity and specificity remain under discussion.

From COMFORT-I onward, TSS50 (the proportion of patients achieving ≥ 50% reduction in TSS from baseline) has emerged as a key binary endpoint for measuring significant symptom improvement. However, the cut-off value defining “significant” improvement warrants further validation, as its clinical sensitivity and specificity remain under discussion. The COMFORT-I trial [33] confirmed the sensitivity of MF-SAF v2.0 and TSS50 to detect clinically significant changes [40]. Ruxolitinib demonstrated clear benefit, with SVR35 achieved in 41.9% versus 0.7% and TSS50 in 45.9% versus 5.3% (ruxolitinib vs. placebo), alongside improvements across QLQ-C30 and PROMIS Fatigue domains [33]. These findings led to ruxolitinib becoming the first MF therapy approved with PRO data included in the label, cementing the regulatory and clinical relevance of TSS.

In fedratinib’s phase III JAKARTA (vs. placebo) [43] and phase II JAKARTA-2 trials [54], a simplified version of MF-SAF v2.0 was used, retaining six core symptoms. Inactivity was removed because it was deemed more indicative of overall functional status than a distinct symptom, introducing conceptual ambiguity. In JAKARTA [43], TSS was calculated from the average of daily symptom scores during the final treatment week and 7-day baseline; in JAKARTA-2 [54], weekly averages (requiring ≥ 5 of 7 days of data) were used. Fedratinib showed significant symptom improvement: TSS50 was achieved by 40.4% of patients versus 8.6% with placebo; SVR35 was achieved by 36% versus 1% [43]. Post hoc analyses further validated its benefit: 23.2% of fedratinib-treated patients experienced clinically meaningful gains in EQ-5D-3L health utility indices, compared to 6.5% with placebo [67]. Despite the FDA approval fedratinib in 2019 based on SVR and clinical benefit, TSS data were not formally cited in the label, underscoring an ongoing challenge in standardizing symptom-based endpoints for regulatory decisions. These trials reinforced the clinical validity and regulatory relevance of TSS, particularly TSS50, in capturing meaningful symptom improvements and aligning symptom burden reduction with HRQoL gains in MF. This evolution of MF-SAF v2.0 marked a shift toward patient-centered efficacy metrics in myelofibrosis trials.

#### Expansion to MPN-SAF and MPN-SAF TSS

To address symptom heterogeneity across PV, ET, and MF, the MPN-SAF was developed in 2011 [51]. It consists of an 18-item core instrument assessing MPNs-specific symptoms (e.g., microvascular disturbances, mood symptoms), a single-item global QoL measure, and is co-administered with the 9-item BFI. The18-item core tool was validated in an international cohort of 402 patients (161 ET, 145 PV and 96 MF), demonstrating excellent reliability (Cronbach’s α ≈ 0.86–0.89), strong construct validity (as evidenced by correlation with the QLQ-C30), and good responsiveness to treatment [51]. Most items use a 7-day recall period, while the BFI fatigue item retained a 24-h recall. To enhance usability, the MPN-SAF was later developed into a 10-item version, the MPN-SAF TSS, also known as MPN-10 [15]. This tool includes nine non-QOL symptoms items from the original MPN-SAF (e.g., concentration, early satiety, inactivity, night sweats, itching, bone pain, abdominal discomfort, weight loss, fever) and one BFI-derived fatigue item “worst fatigue in the past 24 h”. Each item is rated on a 0–10 NRS, generating a total score (0–100), with clinical thresholds (e.g., total score > 20 or individual item > 4) used to stratify symptom burden. By excluding the QoL item, the MPN-SAF TSS focuses exclusively on symptom severity, showing high internal consistency (Cronbach’s α = 0.83) and strong correlations with QLQ-C30 functional scales. Owing to its brevity, reliability, and cross-cultural adaptability, the MPN-SAF TSS has become a standard PROM in clinical trials, especially multinational trials (evaluating jaktinib in China [55]) and observation research [44, 56].

#### Modified MPN-SAF TSS version in later JAK inhibitor trials

Pacritinib, a JAK2/FLT3 inhibitor developed for MF patients with severe cytopenias, was evaluated in the phase III PERSIST-1 [57] and PERSIST-2 [58] trials. Initially, the validated MPN-SAF TSS was used. However, a 2013 protocol amendment introduced the MPN-SAF TSS 2.0 to more accurately reflect MF symptom burden. This version focused on eight core symptoms: tiredness, abdominal discomfort, pain under the ribs on the left side, early satiety, night sweats, pruritus, bone pain, and inactivity. It incorporated the six items from MF-SAF v2.0 alongside “tiredness” and an inactivity item. To ensure consistency, subsequent analyses focused on six overlapping items between the two versions. While recognizing the pivotal role of fatigue in MF, the fatigue item was reintroduced into the TSS assessment, although the fatigue item in MPN-SAF TSS 2.0 retained BFI-style phrasing. Unlike COMFORT-I and JAKARTA, PERSIST-1 [57] and PERSIST-2 [58] enrolled intermediate-1 to high-risk patients, including those with intermediate-1 risk, regardless of baseline platelet or hemoglobin levels. In PERSIST-1, eligibility thresholds evolved from ≥ 3 points in ≥ 2 symptoms (excluding fatigue) using the original MPN-SAF TSS to a total TSS ≥ 13 using the MPN-SAF TSS 2.0. The trial met its primary SVR35 endpoint, with pacritinib demonstrating superior symptom relief: TSS50 was achieved by 25% of intention to treat and 41% of evaluable patients, versus 7% and 10% with best available therapy (BAT). Symptom improvements correlated with PGIC, EQ-5D-5L, and QLQ-C30 scores. In PERSIST-2 (enrolling thrombocytopenic patients), pacritinib again outperformed BAT (including ruxolitinib) for both coprimary endpoints (SVR35 and TSS50). Among ruxolitinib-exposed patients, pacritinib produced higher SVR, numerically greater TSS responses, and greater symptom relief [59]. Nonetheless, its 2022 FDA label did not include PRO-based claims, likely due to PROMs version inconsistencies (MPN-SAF TSS original vs. MPN-SAF TSS 2.0) and lack of formal psychometric revalidation.

The MPN-SAF TSS 2.0 was also utilized in the phase III SIMPLIFY-1 [60] and SIMPLIFY-2 [61] trials of momelotinib, a JAK1/JAK2/activin A receptor type 1 inhibitor. These trials played pivotal roles in exploring momelotinib’s efficacy in different patient populations: SIMPLIFY-1, as the only head-to-head phase III study, directly compared momelotinib with ruxolitinib in JAK inhibitor-naive patients as first-line treatment, while SIMPLIFY-2 evaluated momelotinib against BAT in JAK inhibitor-experienced patients as second-line treatment. The TSS was calculated by summing scores of seven item (inactivity analyzed separately).

In SIMPLIFY-1 [60], momelotinib met the non-inferiority criterion for SVR35 but not for TSS50, though significant anemia-related benefits, including transfusion independence, were observed. In contrast, in SIMPLIFY-2 [61], despite not meeting the SVR35 primary endpoint in JAK inhibitor-experienced patients, exploratory analyses showed that momelotinib led to greater symptom improvement: 26% of patients achieved TSS50 compared to only 6% in the control group, accompanied by improved transfusion independence. A post hoc analysis [46] demonstrated enhanced outcomes, including higher transfusion independence, improved hemoglobin, and symptom/spleen responses, in patients with moderate-to-severe anemia who switched to momelotinib.

While the TSS50 response criterion has long been established as the benchmark for clinical response in MF, the finding from the SIMPLIFY trials initiated a wave of critical re-evaluation. This scrutiny identified a fundamental flaw in the TSS50 standard: it mandates disparate absolute TSS improvements for patients with highly symptomatic (elevated baseline TSS) versus asymptomatic (low baseline TSS) profiles, rendering the threshold inconsistent across different populations. In contrast, anchor-based analyses using the MPN-SAF TSS v2.0 and PGIC revealed that a 32% relative reduction in symptom burden (with absolute thresholds of 8 points in treatment-naive patients and 6 points in pre-treated patients) may better reflect clinically meaningful improvement [62]. This discovery signaled the need for a more refined approach to assessing MF treatment efficacy.

#### Reintroducing fatigue and the emergence of MF-SAF v4.0

The emergence of MF-SAF v4.0 was a significant advancement in MF symptom assessment. Although MF-SAF v2.0, its simplified version, and the modified MPN-SAF TSS version effectively captured TSS changes consistent with HRQoL improvements in JAK kinase inhibitor trials, variability across versions, including item composition, phrasing (e.g., “muscle/bone pain” vs. “bone pain”), recall periods, and response formats, introduced complexity when selecting tools and comparing results across trials. To address this inconsistency, the PRO Consortium’s Myelofibrosis Working Group developed the 7-item MF-SAF v4.0 in 2017 [52], prioritizing the reinstatement of fatigue as a core item and rephrasing it to “average severity” to align with other SAF items. The tool assesses seven core symptoms: fatigue, night sweats, pruritus, abdominal discomfort, pain under the left rib, early satiety, and bone pain. Other key updates included harmonizing item structure (with validated 24-h/7-day recall periods), and removing low-variability items (e.g., fever, weight loss) in line with MF-SAF v2.0. TSS is the sum of seven core items. Importantly, in 2021, the MPN-SAF TSS fatigue item was revised to align with MF-SAF v4.0 [63]. The 2019 MPN Symptom Diary, an electronic platform that mirrors MPN-10 content, enhances remote monitoring and is listed in the APA PsycTests database. Table 1 presents a comparison of symptom assessment items across MPN symptom-specific PROMs.

**Table 1:** Comparison of symptom assessment items across MPN symptom-specific patient-reported outcome measures

Item	MF-SAF [1]	MF-SAF v2.0 [2]	Simplified MF-SAF v2.0 used in JAKARTA trials [3, 4]	MPN-SAF TSS [6]	MPN-SAF TSS 2.0 used in PERSIST [7, 8] and SIMPLIFY [9, 10] trials	MF-SAF v4.0 [11]	2021 Version of MPN-SAF TSS [12]
Fatigue	9-item BFI	X	X	Single item from BFI (worst fatigue, 24 h)	Single item from BFI (worst fatigue, 24 h)	Single item consistent within the SAF (average, 24 h)	Single item consistent within the SAF (average, 24 h)
Fever	√	X	X	√	X	X	√
Weight loss	√	X	X	√	X	X	√
Inactivity	√	Included but not used to compute TSS	X	√	Included but not used to compute TSS	X	√
Abdominal discomfort	√	√	√	√	√	√	√
Pain under the ribs on the left side	√	√	√	X	√	√	X
Pruritus (itching)	√	√	√	√	√	√	√
Night sweats	√	√	√	√	√	√	√
Early satiety (feeling full quickly)	√	√	√	√	√	√	√
Bone pain	√	This item was “Muscle or bone pain”	This item was “Bone or muscle pain”	√	√	√	√
Concentration	X	X	X	√	X	X	√

MF-SAF, Myelofibrosis Symptom Assessment Form; MPN-SAF, Myeloproliferative Neoplasms Symptom Assessment Form; BFI, Brief Fatigue Inventory; TSS, Total Symptom Score

The adoption of MF-SAF v4.0 in subsequent clinical trials marked a paradigm shift. The MOMENTUM trial was the first to use MF-SAF v4.0 for symptom assessment and retain TSS50 as the primary endpoint for evaluating efficacy, shifting the focus from SVR35 to patient-reported symptom burden [34]. The trial enrolled symptomatic (TSS ≥ 10 via MF-SAF v4.0), anemic MF patients post-ruxolitinib, regardless of IPSS risk category, with a platelet threshold > 25 × 10^9^/L. Danazol served as an active comparator to reduce placebo bias. MOMENTUM demonstrated significant and durable improvements in TSS, fatigue, and HRQoL, as assessed by MF-SAF v4.0, QLQ-C30, and PROMIS Short Form 10b [64]. Fatigue scores improvements correlated with TSS reductions and better global health status. The tool demonstrated cross-linguistic validity and robust psychometric performance. Internal consistency (Cronbach’s α > 0.87) and test–retest reliability (ICC > 0.82) further validated its stability [65]. Content validity was confirmed in JAK inhibitor-experienced patients [66]. Table 2 overviews the structures and validation of symptom-specific Patient-Reported Outcome Measures utilized in clinical trials of JAK inhibitors. While the MOMENTUM trial established MF-SAF v4.0 as a regulatory-endorsed tool, its validation in JAK-naïve patients addresses a critical unmet need: unbiased symptom assessment in treatment-naive populations, where baseline symptom burden may differ profoundly from relapsed patients. The MANIFEST trial continued to leverage MF-SAF v4.0 to evaluate patient symptoms while maintaining TSS50 as the pivotal criterion for judging treatment efficacy. MF-SAF v4.0’s sensitivity to early symptom changes in JAK-naïve patients enabled precise quantification of pelabresib-ruxolitinib efficacy (56% TSS50 response at week 24), solidifying its role as a benchmark PROM for MPN clinical trials across all treatment lines. Correlational analyses with PGIC revealed a strong association between patient-perceived worsening and objective TSS deterioration (11.89% higher TSS per PGIC category) [47], suggesting that evaluating TSS as a continuous variable has emerged as a promising approach.

**Table 2:** Overview of the symptom-specific patient-reported outcome measures utilized in clinical trials of JAK inhibitors

Instrument	Year	Target population	Item count	Structure	Includes QoL item	Fatigue measurement	Recall period	Scoring method	ePRO available	Psychometric validation
MF-SAF [1]	2009	MF	20	9 BFI, 9 core MF symptoms, 1 QoL, 1 open-text item)	YES	BFI (peak, 24 h)	BFI: 24 h; others: not specified	Each item scored individually; BFI score = mean of 9 questions	NO	–
MF-SAF v2.0 [2]	2009	MF	7	7 core MF symptoms	NO	NO	24 h	0–10 NRS; TSS = sum of 6 items (scale 0–60, inactivity analyzed separately)	YES	–
Simplified version of MF-SAF v2.0 used in JAKARTA [3, 4] trials	2011–2012	MF	6	6 core MF symptoms	NO	NO	24 h	0–10 NRS. The TSS = sum of 6 items (scale 0–60)	YES	–
MPN-SAF [5]	2011	PV, ET and MF	28	18 core MPN symptoms, 1 QoL, 9-item BFI)	YES	BFI (peak, 24 h)	7 days (BFI: 24 h)	Each item scored individually; BFI score = mean of 9 questions	YES	18-item core tool showing Internal consistency (Cronbach’s α ≈ 0.86–0.89) Construct validity (via correlation with QLQ-C30)
MPN-SAF TSS [6]	2012	PV, ET and MF	10	9 items from MPN-SAF + 1 BFI fatigue (worst fatigue)	NO	Single item (BFI: worst fatigue)	7 days (BFI: 24 h)	0–10 NRS. TSS = sum of 10 items (scale 0–100)	YES	Internal consistency (Cronbach’s α = 0.83). Construct validity (via correlation with QLQ-C30)
MPN-SAF TSS 2.0 used in PERSIST [7, 8] and SIMPLIFY [9, 10] trials	2013–2014 2013–2016	MF	8	7 core MF symptoms + 1 BFI fatigue (worst fatigue)	NO	Single item (BFI: worst fatigue)	7 days (BFI: 24 h)	0–10 NRS. TSS = sum of 7 items (scale 0–70, inactivity analyzed separately)	YES	–
MF-SAF V4.0 [11]	2017	Initially used for MF patients, it is increasingly applied to PV and ET	7	7 core MF symptoms	NO	Single item consistent with the SAF (average, 24 h)	24 h or 7 days	0–10 NRS. TSS = sum of 7 items (scale 0–70)	YES	Internal consistency (Cronbach’s α > 0.87) Test–retest reliability (ICC > 0.82)
2021 Version of MPN-SAF TSS [12]	2021	PV, ET and MF	10	9 MPN-SAF symptoms + 1 fatigue item (average)	NO	Single item consistent with the SAF (average, 24 h)	24 h	0–10 NRS. TSS = sum of 10 items (scale 0–100)		Cronbach’s α = 0.89

QoL, Quality of life; PRO, Patient-Reported Outcome; MF-SAF, Myelofibrosis Symptom Assessment Form; MF, Myelofibrosis; BFI, Brief Fatigue Inventory; NRS, Numerical Rating Scale; TSS, Total Symptom Score; QLQ-C30, Quality-of-life questionnaire-core 30; MPN-SAF, Myeloproliferative Neoplasms Symptom Assessment Form; PV, Polycythemia Vera; ET, Essential Thrombocythemia; MPN, Myeloproliferative neoplasms; ICC, Intraclass Correlation Coefficient

Beyond MF, MF-SAF v4.0 has proven its versatility in other MPNs. In ET, it has been used in a phase III study comparing bomedemstat with hydroxyurea [67], as well as the MK-3543-006 trial for ET patient intolerant or refractory to hydroxyurea (NCT06079879). Similarly, in PV, the phase III VERIFY trial (NCT05210790) utilizes MF-SAF v4.0 to assess outcomes in phlebotomy-dependent PV patients receiving standard care, evaluating the hepcidin mimetic peptide therapy Rusfertide against placebo.

In 2023, the FDA approved momelotinib for MF, citing MF-SAF v4.0-based symptom improvement (TSS50) in the label—the first MF therapy approved with a symptom-based PRO as the primary efficacy endpoint.

### Future directions

####  Clinical integration and guideline recommendations

MPNs-symptom-specific PROMs, particularly the MPN-10, are now widely integrated into routine clinical practice. According to consensus guidelines from the International Working Group-Myeloproliferative Neoplasms Research and Treatment [68] and the European LeukemiaNet, a symptom response is defined as TSS50, although smaller improvements may also carry clinical significance. The 2025 National Comprehensive Cancer Network guidelines for MPNs recommend routine MPN-SAF TSS administration at baseline and every 3–6 months to monitor disease activity, treatment response, and patient well-being. In clinical practice, MPNs-symptom-specific PROMs guide risk-adapted therapeutic strategies. In low-risk MF, TSS scores informs whether to observe or initiate treatment (e.g., ruxolitinib, hydroxyurea) in symptomatic patients. In high-risk disease, scores guide JAK inhibitors selection (e.g., momelotinib, pacritinib) or prompt referral for allogeneic stem cell transplantation.

Despite broader access to JAK inhibitors and greater PROM awareness, 40–45% of patients still report worsening symptoms over time (PV: 47%, ET: 40%, MF: 45%) [16], highlighting a persistent gap in symptom control.

#### Unmet challenges in symptom burden management

Although PROMs are guideline-endorsed and clinically validated, their uptake in routine clinical practice remains low. The MPN Landmark survey found that only 26% of physicians systematically used validated symptom assessment forms [11]. This disconnect is driven by workflow integration issues, limited clinician training, and continued reliance on hematologic parameters. To bridge this gap, PROMs must be embedded into electronic health records, prompted at key decision points, and supported by ongoing clinician education.

Despite their regulatory and clinical importance, current PROMs still face critical limitations in two key areas. First, the emergence of novel JAK inhibitors, targeted therapies, and combination regimens has led to increasingly diverse symptom profiles in MPNs. Side effects such as anemia, gastrointestinal discomfort, and fatigue vary across treatment modalities. Moreover, treatment adherence, a major driver of symptom control, is rarely captured in existing PROMs. The ROMEI study showed that between 25 and 40% of patients on ruxolitinib exhibited low to moderate adherence, often unintentionally, when evaluated using the Morisky Medication Adherence Scale, a tool widely adopted in chronic disease settings [68]. Future PROMs should incorporate modular designs that accommodate therapy-specific toxicities and integrate adherence assessment into routine symptom monitoring.

Second, symptom burden in MPNs is influenced by underlying disease biology. For example, JAK2V617F allele has been associated with symptom severity, including pruritus [13, 69]. Additionally, elevated C-reactive protein and lactate dehydrogenase levels have been linked to higher symptom scores in PV and MF [16]. However, current PROMs remain largely disconnected from these molecular indicators. To fully realize personalized symptom management, future PROMs should integrate longitudinal biomarker data with patient-reported outcomes, enabling more biologically informed assessments and interventions.

## Conclusion

MPNs are fundamentally symptom-driven malignancies, where accurately capturing and managing symptom burden is central to improving both HRQoL and treatment outcomes. Over the past decade, the evolution of MPN-symptom-specific PROMs, most notably the MF-SAF series, has enabled more structured, sensitive, and clinically actionable symptom assessment. These tools have become indispensable in both clinical trials and real-world care, guiding therapeutic decisions and supporting regulatory approvals.

Equally significant is the refinement of TSS-based endpoints. TSS50 has become a widely accepted standard in trials; however, its binary nature may obscure subtler yet clinically meaningful changes. Emerging evidence supports the use of TSS as a continuous variable, offering improved granularity and responsiveness in efficacy evaluation. Taken together, the dual progression of PROM methodology and endpoint sophistication underscores a paradigm shift: from symptom measurement as a supportive element to its recognition as a core pillar of precision, patient-centered care in MPNs. Continued innovation in both domains will be key to optimizing symptom control and long-term outcomes for patients.

## Data Availability

No datasets were generated or analysed during the current study.

## Abbreviations

PROs: Patient-reported outcomes
FDA: Food and drug administration
PROMs: Patient-reported outcome measures
HRQoL: Health-related quality of life
MPNs: Myeloproliferative neoplasms
PV: Polycythemia vera
ET: Essential thrombocythemia
PMF: Primary myelofibrosis
MF: Myelofibrosis
JAK: Janus kinase
BFI: Brief fatigue inventory
FACT: Functional assessment of cancer therapy
EORTC: European Organization for Research and Treatment of Cancer
QLQ-C30: Quality-of-life questionnaire-core 30
GSG-MPN: German Study Group MPN Bioregistry
MF-SAF: Myelofibrosis symptom assessment form
MPN-SAF: MPN symptom assessment form
TSS: Total symptom score
IPSS: International Prognostic Scoring System
DIPSS: Dynamic International Prognostic Scoring System
OS: Overall survival
HM-PRO: Hematological malignancy-specific patient-reported outcome
PROMIS: The patient-reported outcomes measurement information system
EQ-VAS: EQ Visual Analog Scale
SF-36: The Short Form 36-Item Health Survey
PGIC: The patient global impression of change
COSMIN: Consensus-based standards for the selection of health measurement instruments
NRS: Numeric rating scale
BAT: Best available therapy
